# Gold and silver nanoparticles in Alzheimer's and Parkinson's diagnostics and treatments

**DOI:** 10.1002/ibra.12126

**Published:** 2023-08-14

**Authors:** Edoardo Scarpa, Mariafrancesca Cascione, Anna Griego, Paolo Pellegrino, Giorgia Moschetti, Valeria De Matteis

**Affiliations:** ^1^ Department of Pharmaceutical Sciences (DISFARM) University of Milan Milan Italy; ^2^ Infection Dynamics Laboratory‐National Institute of Molecular Genetics (INGM) Milan Italy; ^3^ Department of Mathematics and Physics “Ennio De Giorgi” University of Salento Lecce Italy; ^4^ National Research Council of Italy (CNR)‐Institute for Microelectronics and Microsystems (IMM) Lecce Italy

**Keywords:** blood–brain barrier, metallic nanoparticles, nanomedicine, neurodegenerative diseases

## Abstract

Neurodegenerative diseases (NDs) impose substantial medical and public health burdens on people worldwide and represent one of the major threats to human health. The prevalence of these age‐dependent disorders is dramatically increasing over time, a process intrinsically related to a constantly rising percentage of the elderly population in recent years. Among all the NDs, Alzheimer's and Parkinson's are considered the most debilitating as they cause memory and cognitive loss, as well as severely affecting basic physiological conditions such as the ability to move, speak, and breathe. There is an extreme need for new and more effective therapies to counteract these devastating diseases, as the available treatments are only able to slow down the pathogenic process without really stopping or resolving it. This review aims to elucidate the current nanotechnology‐based tools representing a future hope for NDs treatment. Noble metal nano‐systems, that is, gold and silver nanoparticles (NPs), have indeed unique physicochemical characteristics enabling them to deliver any pharmacological treatment in a more effective way within the central nervous system. This can potentially make NPs a new hope for reversing the actual therapeutic strategy based on slowing down an irreversible process into a more effective and permanent treatment.

## Graphical abstract

Gold nanoparticles (AuNPs) and silver nanoparticles (AgNPs) are emerging as promising tools in the application for treating neurodegenerative diseases. Thanks to their unique properties, these nanoparticles (NPs) can be used for various purposes in this field of medicine. In the last years, more efforts are involved in the application of NPs as new agents both for early diagnosis and therapy of neurodegenerative diseases, thanks to their ability to overcome the Blood–Brain Barrier. Their unique physicochemical properties make them powerful tools for the development of a new nanoplatform to contrast Alzheimer's and Parkinson's diseases.

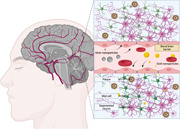

## 1 INTRODUCTION

Nanotechnology has emerged as a promising field in the treatment of neurodegenerative disorders such as Alzheimer's disease (AD) and Parkinson's disease (PD). Among the different nanoparticles (NPs) being explored for therapeutic applications, gold nanoparticles (AuNPs) and silver nanoparticles (AgNPs) have gained significant attention due to their unique properties and potential benefits in combating these disorders.^1^ AuNPs and AgNPs possess several characteristics that make them suitable for targeted drug delivery and imaging in neurological diseases. Their small size, large surface area‐to‐volume ratio, and tunable surface chemistry enable the efficient crossing of the blood–brain barrier (BBB), which is a major challenge in delivering therapeutic agents to the brain.^2^ Additionally, AuNPs and AgNPs can be functionalized with specific ligands or antibodies to enhance their selectivity toward diseased cells or biomarkers associated with Alzheimer's and Parkinson's.^3^ In the context of AD, these NPs have shown promise in reducing the aggregation and toxicity of amyloid‐beta (Aβ) plaques, which are hallmark pathological features of the disease. By binding to Aβ peptides and inhibiting their aggregation, these NPs can potentially prevent the formation of toxic oligomers and fibrils, thereby slowing down the progression of Alzheimer's pathology.^4^ Furthermore, the unique optical properties of AuNPs enable their use in diagnostic techniques, such as surface‐enhanced Raman spectroscopy, for the early detection and monitoring of Alzheimer's‐related biomarkers.^4^ Similarly, in the case of PD, noble metals have demonstrated their potential in targeting misfolded alpha‐synuclein protein aggregates, which are characteristic of the disease.^5^ By interfering with the aggregation process and promoting the clearance of these protein aggregates, AuNPs, and AgNPs could help alleviate the neurotoxicity associated with Parkinson's. Additionally, their ability to generate localized heat through photothermal therapy opens new possibilities for noninvasive treatment approaches in PD.^6^

Despite the promising advancements, it is important to acknowledge that the application of AuNPs and AgNPs in the treatment of AD and PD is still in its early stages. Further research is needed to address concerns related to NP stability, biocompatibility, and long‐term effects. The focus of this review is to discuss their potential in the treatment and diagnosis of ND diseases with different approaches.

## 2 NEURODEGENERATION

**Figure 1:**
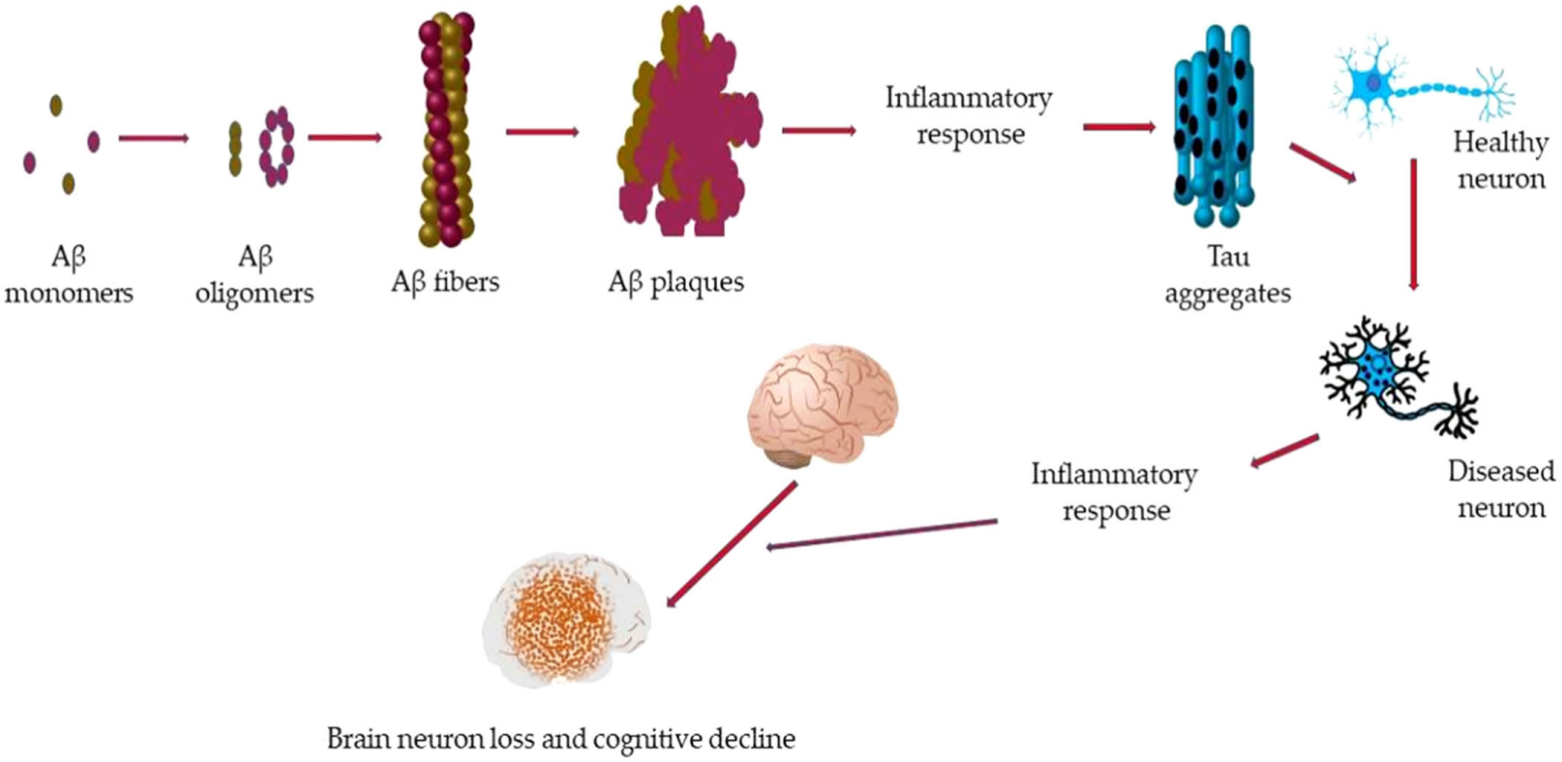
Schematic representation of cognitive decline in Alzheimer's disease. Amyloid‐beta (Aβ) monomers aggregate forming variant structures that, following aggregation phenomena, create Aβ fibers, forming Aβ plaques inducing inflammation. The latter led to the τ aggregates inducing neuron damage. The increase of damaged neurons induces the onset of neuron cell loss and cognitive impairment.^13^ [Color figure can be viewed at wileyonlinelibrary.com]

Neurons are specialized cells found mainly in the brain. During the childhood period, neural stem cells progenitors produce most of the neurons and their number drastically reduces with aging.^7^ As the average life span of the world's population has enhanced due to improved medical treatments and quality of life, the prevalence of senescence‐related diseases has increased in recent years.^8^ Nonetheless, neurons in the brain can undergo progressive degeneration and dysfunction due to the onset of various disorders that are generally grouped under the umbrella of neurodegenerative diseases (NDs). These conditions primarily affect the structure and function of the brain, leading to a decline in cognitive abilities,^9^ movement control, and overall neurological function.^10^ At the tissue level, most of the NDs are characterized by the breakdown of the BBB, which starts at the initial phase of neurodegeneration inducing functional and structural changes in the microvasculature,^11^ as well as other molecular alterations such as mitochondrial dysfunction, oxidative stress, and dysfunctional transport mechanisms promoting incorrect neural signaling and ions unbalance.^12^ Among all the neurodegenerative diseases, the most common across this and the last century are indeed AD and PD, which will be the focus of this review.^13^ Despite being different pathologies, both AD and PD are characterized by a massive accumulation of altered proteins (extracellular or intracellular) in the brain^14^ (Figure 1). Despite the onset of such pathologies still unknown, the scientific community is agreeing that factors such as genetic control, lifestyle, and environment have a crucial role. Importantly, the current therapies for these diseases aim at mitigating the symptoms concerning cognitive and physical decline rather than acting on the molecular mechanisms.^15^

### 2.1 AD

Alzheimer's is the most common chronic and progressive neurodegenerative disease inducing memory loss and impairing normal daily activities. AD induces dementia in the old‐age population of developed countries it is estimated that about 5% of the population over 65 and about 20% of people with an age greater than 85 are affected by AD.^16^ In addition, there are some cases in which the effects of AD start even among 50‐year‐old patients.^17^ AD brings to hippocampus degeneration, which in turn leads to the alteration of synaptic connection due to the formation of plaques constituted by amyloid proteins. At the molecular level, the brain tissue in AD is characterized by the combined presence of two classes of abnormal, insoluble, and highly dense structures: extracellular amyloid plaques and intraneuronal neurofibrillary tangles. The soluble component of these structures is the Aβ peptides for plaques and tau (τ) proteins for tangles. Aβ peptides derive from amyloid precursor protein (APP), which in healthy individuals is normally processed and cleared from the brain. However, in AD, Aβ accumulates abnormally, leading to the formation of insoluble plaques between neurons. Aβ is believed to be one of the main factors initiating the pathological cascade in AD.^18^ τ protein is a microtubule‐associated protein that helps stabilize and support the structure of neurons. In AD and other tauopathies, τ protein becomes hyperphosphorylated, triggering the creation of insoluble and twisted neurofibrillary tangles that accumulate within neurons impairing their function. During the past dozen years, a steadily accumulating body of evidence has indicated that soluble forms of Aβ and τ work together, independently of their accumulation into plaques and tangles, to drive healthy neurons into the diseased state. Aβ is upstream of τ in AD pathogenesis and triggers the conversion of τ from a normal to a toxic state.^19^

At present, AD is treated by a combination of several drugs, including acetylcholinesterase inhibitors (donepezil, galantamine, and rivastigmine) that reduce the symptoms such as depression and general behavioral imbalance. Acetylcholinesterase inhibitors improve some cognitive symptoms (such as memory and attention) and behavioral (such as apathy, agitation, and hallucinations), but they are not capable of stopping the progression of the disease.^20^ Another treatment consists of the use of memantine, indicated in moderately severe and severe AD. Memantine works by moderating the toxic effects deriving from the excessive excitation of nerve cells caused by glutamate: it has an essential role in learning and memory, but its excess produces an abnormal amount of calcium in the nerve cells.^21^

### 2.2 PD

**Figure 2:**
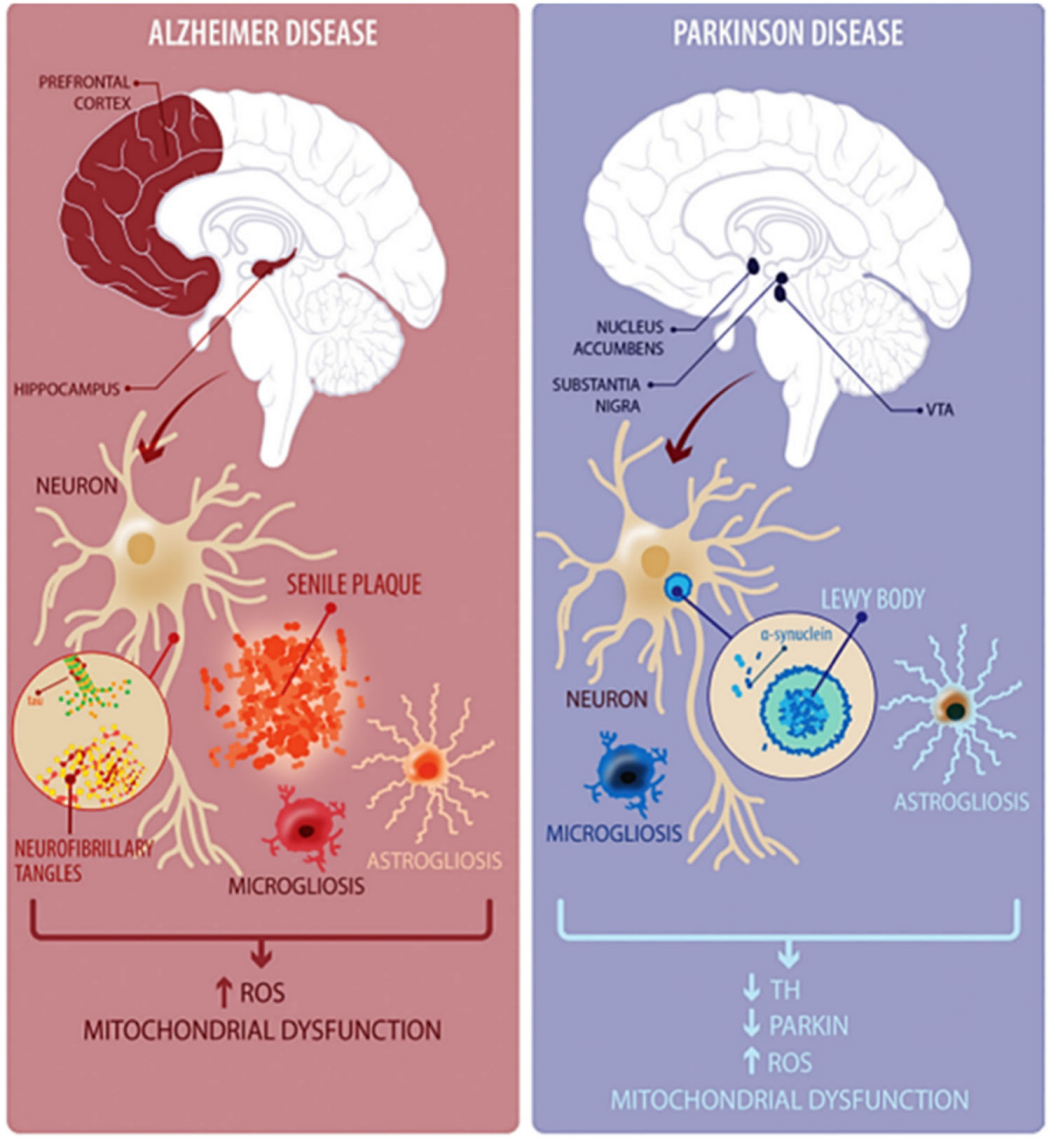
Pathological milieu of Alzheimer diseases (ADs) and Parkinson's diseases (PDs). In AD the most important phenomenon is the amyloid‐β aggregates (senile plaques) formation and neurofibrillary tangles constituted by hyperphosphorylated τ protein. Contrary, in PD progression, in substantia nigra the loss of dopaminergic circuitry is the main feature with the formation of Lewy bodies and neurites. The latter are composed of α‐synuclein (SNCA) aggregates. In both diseases, reactive oxygen species production and mitochondrial dysfunction are observed.^29^ [Color figure can be viewed at wileyonlinelibrary.com]

PD is evinced in people over 50 with a preference for men over women. PD induces atrophy of the frontal cortex and ventricular expansion as well as nerve cell loss in substantia nigra pars compacta (SNpc), a neuronal formation of the midbrain, located in the cerebral peduncles, involved in the execution of several motor functions.^22^ This alteration triggers the death of dopaminergic (DA) neuromelanin‐containing neurons, inducing dysfunction of the nigrostriatal pathway that leads to a decrease in dopamine concentration.^23^ This deficit is manifested with the typical symptoms of PD, that is, tremors, muscle stiffness, and difficulty in walking. Among the biochemical mechanisms involved in PD progression, the most important are the α‐synuclein misfolding and aggregation, dysfunctional autophagy–lysosome system, and mitochondrial damage.^24^ In particular, α‐Syn is a small protein encoded by the SNCA gene, and although its exact function remains largely unknown, it is believed that α‐Syn is involved in synaptic plasticity and neurotransmitter release. In physiological conditions, α‐Syn is in a dynamic equilibrium between unfolded monomers and α‐helically folded tetramers.^25^ However, an imbalance in the tetramer: monomer ratio can lead to the preponderance of aggregating forms evolving from oligomers to polymers that could converge into fibrous filaments in the form of Lewy bodies and Lewy neurites^19^ As previously reported for AD, also in PD τ protein misfolding occurs, which leads to plaques formation.^24^ The main therapy for PD is levodopa (L‐3,4‐dihydroxyphenylalanine or l‐dopa), an intermediate amino acid in the dopamine biosynthetic pathway. Levodopa can cross the BBB and, upon reaching the brain, it is absorbed by nerve cells and transformed into dopamine. The aim of this treatment is to supply the brain with dopamine, reducing the collateral effects due to its shortage.^26^ The life quality of patients can be improved also by brain stimulation. Peripheral dopa‐decarboxylase inhibitors, such as carbidopa, can be administered in combination with levodopa to improve its absorption and passage through the central nervous system (carbidopa prevents levodopa from being transformed into dopamine before reaching the brain).^27^ However, as for AD, the progression of the disease can be slowed but never stopped really.^28^ A schematic pathological milieu of AD and PD is reported in Figure 2.

## 3 THE BBB PHYSIOLOGY

**Figure 3:**
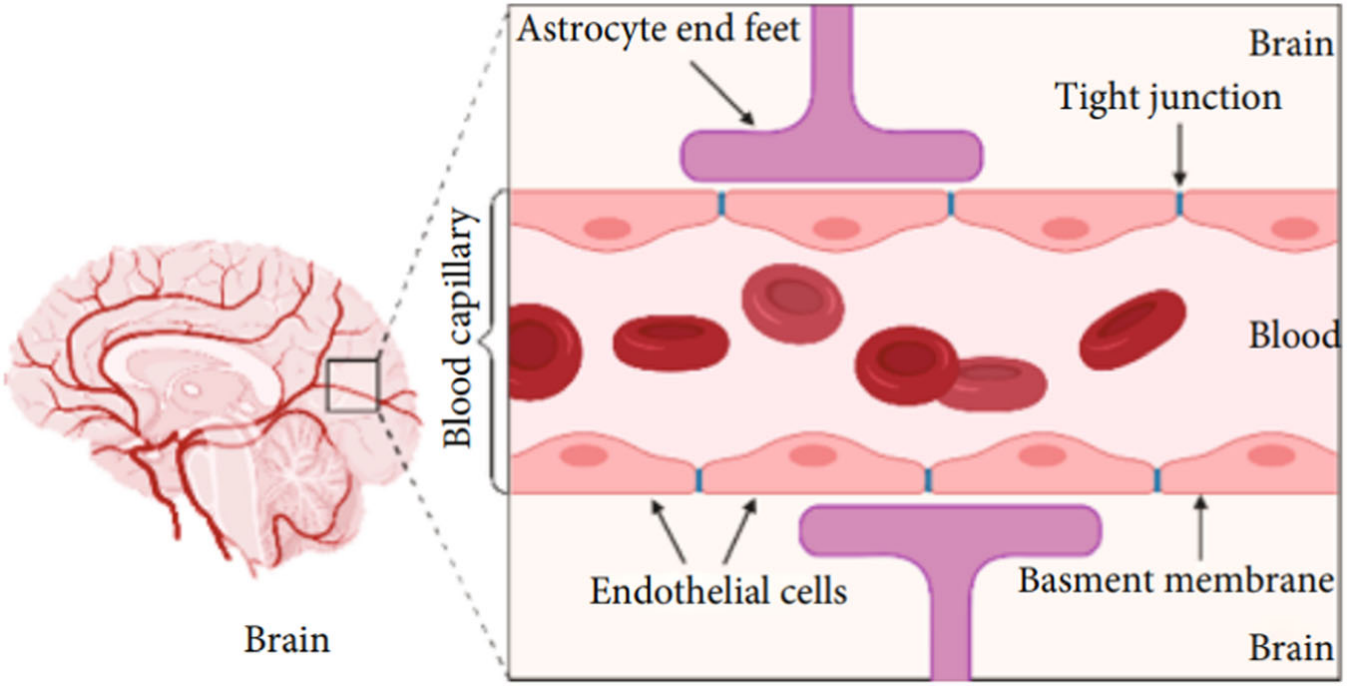
Anatomy of blood–brain barrier.^30^ The main cell types are astrocytes and endothelial cells characterized by high concentrations of tight junctions. [Color figure can be viewed at wileyonlinelibrary.com]

The BBB is a diffusion barrier that physically separates the brain from the circulatory system.^30^ Its main function is to protect the brain from toxins and pathogens and, at the same time, to facilitate the useful molecules to reach the brain parenchyma.^31^ BBB consists mainly of endothelial cells joined together by *tight junctions* which prevent the crossing of chemical species with high molecular weight.^32^ Unlike the other endothelial cells characterizing the capillaries, the junctions are narrower, for enhancing the filtering ability that is greater than other body districts.^32^ The endothelium of the cerebral capillaries is therefore continuous and without fenestrations, indispensable requirements for the protection of neurons and of the cerebral tissue in general.^31^ The astrocytic extensions also contribute to the formation of the BBB, the presence of which further insulates the nervous tissue and increases the filtering capacity of the barrier itself^33^ (Figure 3). The astrocytic processes, in fact, end with swellings, called pedicels or peduncles, which affix themselves to the capillaries of the BBB. Arranged in regular rows along the blood vessels, they avoid direct contact between the vessel wall and the surrounding nervous tissue, further preventing the flow of nonlipophilic or high pm substances from the blood to the intermittent space and there to the nerves.

This particular arrangement of astrocytic processes is called glia limitans.^34^ Astrocytes are in direct contact with the cerebral capillaries forming the BBB, which allows them to regulate: (i) the formation of the tight junctions between the endothelial cells of the BBB by releasing transforming growth factor (TGFα) and glial‐derived neurotrophic factor (GDNF); (ii) the flow and pressure of cerebral blood vessels; (iii) the encephalic homeostasis of water through aquaporins; (iv) the chemical content of the extracellular space; (v) the diffusion of neurotransmitters; and (vi) the extracellular concentration of many substances that can interfere with the correct neuronal function (e.g., potassium ions).^35^, ^36^, ^37^ Only essential molecules such as amino acids, oxygen, and water can freely access the brain, whereas others like glucose can only enter by active protein‐mediated transport.^38^ Again, neutral lipophilic molecules can pass the BBB by passive diffusion mechanism, whereas molecules having a molecular weight greater than 600 Da are not able to cross BBB.^39^ The restrictive nature of the BBB is a physical barrier to drug delivery to the brain. When designing a drug for the brain, the possibility of the BBB blocking the molecule must be considered creating methods that bypass the BBB obstacle.^31^, ^40^ The integrity of the BBB is influenced by numerous factors, among these the state of health and the age of the subject. An inflammatory state, for example, caused by a bacterial infection (meningitis), weakens the BBB, resulting in reduced selectivity.^41^

The crossing of the BBB involves a combination of passive and active transport mechanisms, tightly regulated to maintain brain homeostasis and protect the central nervous system from potential harm.^42^ Understanding these mechanisms is crucial for developing targeted therapies and drugs for various neurological conditions. The crossing of the BBB is a complex process involving various mechanisms. The first mechanism is passive diffusion, where small lipophilic molecules, such as oxygen, carbon dioxide, and some lipid‐soluble drugs, can passively diffuse across the BBB due to their ability to dissolve in the lipid bilayer of endothelial cells that form the barrier.^43^ The second route is transcellular transport where some small molecules, including glucose and amino acids, can use specific transporters located on the surface of brain endothelial cells to facilitate their transport across the barrier. These transporters allow the selective movement of essential nutrients into the brain.^44^ The third way of transport is carrier‐mediated transport: larger molecules, such as neurotransmitters and metabolic substrates, rely on carrier‐mediated transport systems to cross the BBB.^45^ These transporters facilitate the movement of substances through the endothelial cells, ensuring their controlled and specific passage. Large molecules, like insulin, can cross the BBB through receptor‐mediated transcytosis triggering internalization and transport of the complex across the cells.^46^ Certain peptides and proteins, characterized by positively charged residues, can cross the BBB by adsorptive transcytosis. They interact with negatively charged components of the endothelial cell membrane, facilitating their transcellular transport.

Starting from these, inorganic NPs, such as AuNPs and AgNPs, can potentially cross the BBB and gain access to the brain's neural tissue.^47^ This process relies on several key mechanisms such as passive diffusion and receptor‐mediated transcytosis. The first is related to small NPs with appropriate surface properties, particularly those with hydrophobic coatings, which can passively diffuse across the BBB. Their small size allows them to traverse the lipid bilayer of endothelial cells that form the barrier. The second is associated with larger inorganic NPs can use receptor‐mediated transcytosis.^48^ This involves NP binding to specific receptors on the surface of brain endothelial cells, triggering internalization of the NP‐receptor complex and transport across the cells. In addition, some inorganic NPs can exploit adsorptive transcytosis to cross the BBB. Positively charged NPs interact with negatively charged components of the endothelial cell membrane, leading to their transcellular transport. To improve the NPs cross, their surface can be functionalized by attaching specific ligands or functional groups to the NPs.^49^

It is essential to note that the transport of inorganic NPs across the BBB is still an area of active research, and the exact mechanisms can vary depending on NP properties, such as size, and surface modifications. The effectiveness of this barrier obviously depends on the maintenance of its biochemical, biophysical, anatomical, and functional characteristics.^41^ Then, neurological diseases are characterized by modification of BBB functions. The BBB dysfunction can induce ion unbalance and the immune cells uptake in the brain that can produce degeneration of neurons. The BBB damage at endothelial and astrocytes level is associated with AD and PD onset.^30^

The breakdown of the BBB in neurodegenerative diseases can have significant implications for disease progression.^50^ One mechanism by which neurodegenerative diseases contribute to BBB disruption is through the accumulation of toxic protein aggregates. Proteins such as Aβ and τ, have been shown to directly affect the BBB's function. These aggregated proteins can induce inflammation and oxidative stress, leading to damage of the endothelial cells that line the blood vessels in the brain. This damage compromises the tight junctions between these cells, allowing for increased permeability of the BBB.^51^

In addition to protein aggregation, neuroinflammation is another hallmark of neurodegenerative diseases that can contribute to BBB breakdown.^52^ The proinflammatory molecules can disrupt the tight junctions and compromise the integrity of the BBB. The chronic inflammation observed in neurodegenerative diseases further amplifies this effect, perpetuating a cycle of BBB dysfunction. Furthermore, the accumulation of misfolded proteins and the ensuing neuroinflammation can activate matrix metalloproteinases (MMPs), enzymes that degrade the extracellular matrix components of the BBB. MMPs can break down the basement membrane and disrupt the tight junction proteins, leading to increased permeability of the BBB.^53^

The compromised BBB in neurodegenerative diseases allows the entry of harmful molecules, such as toxins and immune cells, into the brain. This influx can further exacerbate inflammation and neuronal damage, contributing to disease progression.^2^ Understanding the mechanisms by which neurodegenerative diseases disrupt the BBB is crucial for developing therapeutic strategies aimed at preserving its integrity. Targeting the factors that contribute to BBB breakdowns, such as protein aggregation, neuroinflammation, and MMP activation, could potentially help restore BBB function and provide neuroprotection in these diseases.

## 4 NANOTECHNOLOGY FOR NDS

The advent of nanotechnology has opened up several avenues in different fields, particularly in nanomedicine.^54^ Due to the unique properties of NPs, they can be used as powerful contrast agents for diagnostics or as carriers for drug delivery.^55^ Indeed, thanks to the possibility of functionalizing their surface or confining the biologically active molecules within them, they can reach a specific site in the organism, thus maximizing conventional therapy.^56^, ^57^ In the context of neurodegenerative diseases, the use of NPs is particularly attractive, due to their size between 1 and 100 nm that allows them to penetrate the BBB.^58^ Particle size plays thus a key role in the biodistribution of NPs.^2^ After administration, the NPs enter the blood system reaching different kinds of organs. In general, the smaller NPs with a diameter of about 5–10 nm are rapidly excreted by renal function, whereas the NPs with a bigger size are accumulated in the spleen, heart, stomach, and liver.^59^, ^60^ In addition to size, also the surface properties of NPs influence their distribution and uptake in cells. NPs having a positive charge tend to induce aggregation due to their bond with serum proteins that are negatively charged.^61^ Positively charged particles can form aggregates in the presence of negatively charged serum proteins, thus inducing dangerous effects such as embolism in lung capillaries.^62^ The blood–cephalic barrier, which as we reported in the previous paragraphs, is characterized by the presence of thigh junctions, is a natural barrier for larger NPs. Conversely, NPs that are very small in size can cross the barrier reaching the brain, similarly to small biological molecules. Clearly, in case NPs are used for therapeutic purposes, this can be a significant advantage. In contrast, in the case where the body is exposed to very small toxic NPs, these can cause neurotoxicity phenomena as is the case in highly polluted areas of the world.

Noble metal NPs such as AuNPs and AgNPs are characterized by high stability, easy synthetic routes, and tunable surface functionalization. Au and AgNPs have shown potential in preventing the aggregation of extracellular and intracellular protein aggregates in ND. Firstly, Au and AgNPs can act as stabilizers and inhibit the misfolding of proteins by binding to specific regions of the protein molecules. This interaction prevents the proteins from adopting abnormal conformations and subsequently forming toxic aggregates.^63^ Second, noble metal NPs can serve as “seeds” for protein aggregation. By presenting a surface that mimics the protein's native structure, they attract the misfolded proteins and facilitate their binding to the NP surface. This sequesters the proteins away from other molecules, reducing their ability to aggregate with each other and form larger aggregates. Furthermore, Au and AgNPs possess excellent catalytic properties. They can promote the degradation of misfolded proteins through catalytic reactions, such as the generation of reactive oxygen species or the activation of cellular enzymes involved in protein clearance pathways. This enhanced protein degradation helps prevent the accumulation of toxic protein aggregates.^64^ When appropriately functionalized, these NPs can selectively interact with inflammatory cells in the brain, thereby attenuating the neuroinflammatory response.^65^, ^66^ Since an inflammation state is persistent in NDs^67^ AuNPs and AgNPs have shown intrinsic antioxidative properties,^68^ capable of scavenging reactive oxygen species (ROS), which play a pivotal role in neuroinflammation‐mediated neuronal damage. By reducing ROS levels, these NPs may ameliorate oxidative stress‐induced inflammation in the brain. In addition, Au and Ag NPs have been reported to inhibit the production and release of proinflammatory cytokines, such as interleukin‐1β (IL‐1β), tumor necrosis factor‐alpha (TNF‐α), and IL‐6,^69^, ^70^ which are crucial contributors to neuroinflammation. By suppressing these cytokines, NPs may dampen the inflammatory cascade in the brain. In addition, Au and AgNPs are involved in nuclear factor‐kappa B (NF‐κB) pathway inhibition. NF‐κB is a key transcription factor responsible for initiating the expression of numerous proinflammatory genes.^71^ NPs interfere with the NF‐κB signaling pathway, thus suppressing the production of inflammatory mediators.^72^

**Figure 4:**
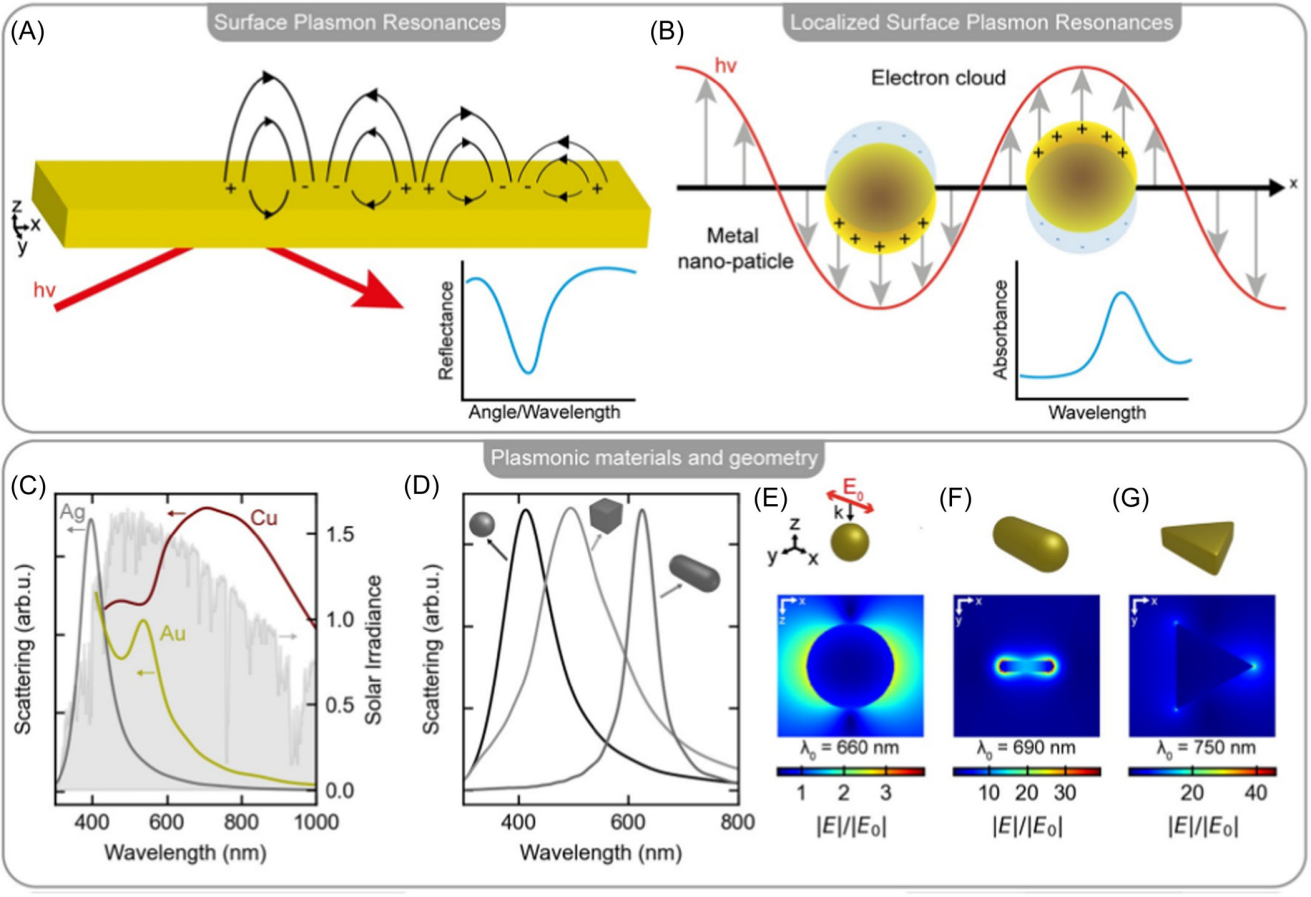
(A) Surface plasmon resonance (SPR) of bulk gold (B) Schematic representation of a localized surface plasmon resonance (LSPR) characterized by electron oscillation when light reaches the nanometal interface. (C) LSPR resonances for spherical Au, Ag, and Cu nanomaterials (D) Size and shape dependence of LSPR for Ag nanostructures. (E) Au sphere, radius: 90 nm. (F) Au rod, length: 76 nm radius 8 nm. (G) Au triangle, side length 105 nm.^77^ [Color figure can be viewed at wileyonlinelibrary.com]

AuNPs are metallic NPs that are the most widely used in medicine.^73^ In fact, they are chemically inert since in most cases, they do not undergo oxidation processes, and their surface can be easily functionalized with different types of chemical groups or dyes.^74^ The distinguishing property of AuNPs is surface plasmon resonance (SPR), an optical phenomenon occurring when light irradiates the metal surface.^75^ This interaction triggers the oscillation of valence electrons occurring between metal and dielectric (e.g., air) resulting in characteristic absorption peaks.^76^ Same behavior is manifested in silver and copper. These absorptions depend on the size and shape of the NPs. Therefore, it is possible to shift from the visible to the infrared by modulating the NPs size and shape (Figure 4).^77^ In the context of neurodegenerative diseases, several studies have shown that the use of AuNPs can help both as a contrast and as a therapeutic agent, due in part to the ability of the smaller‐sized NPs to cross the BBB.^54^

### 4.1 AuNPs as therapeutic agents

**Figure 5:**
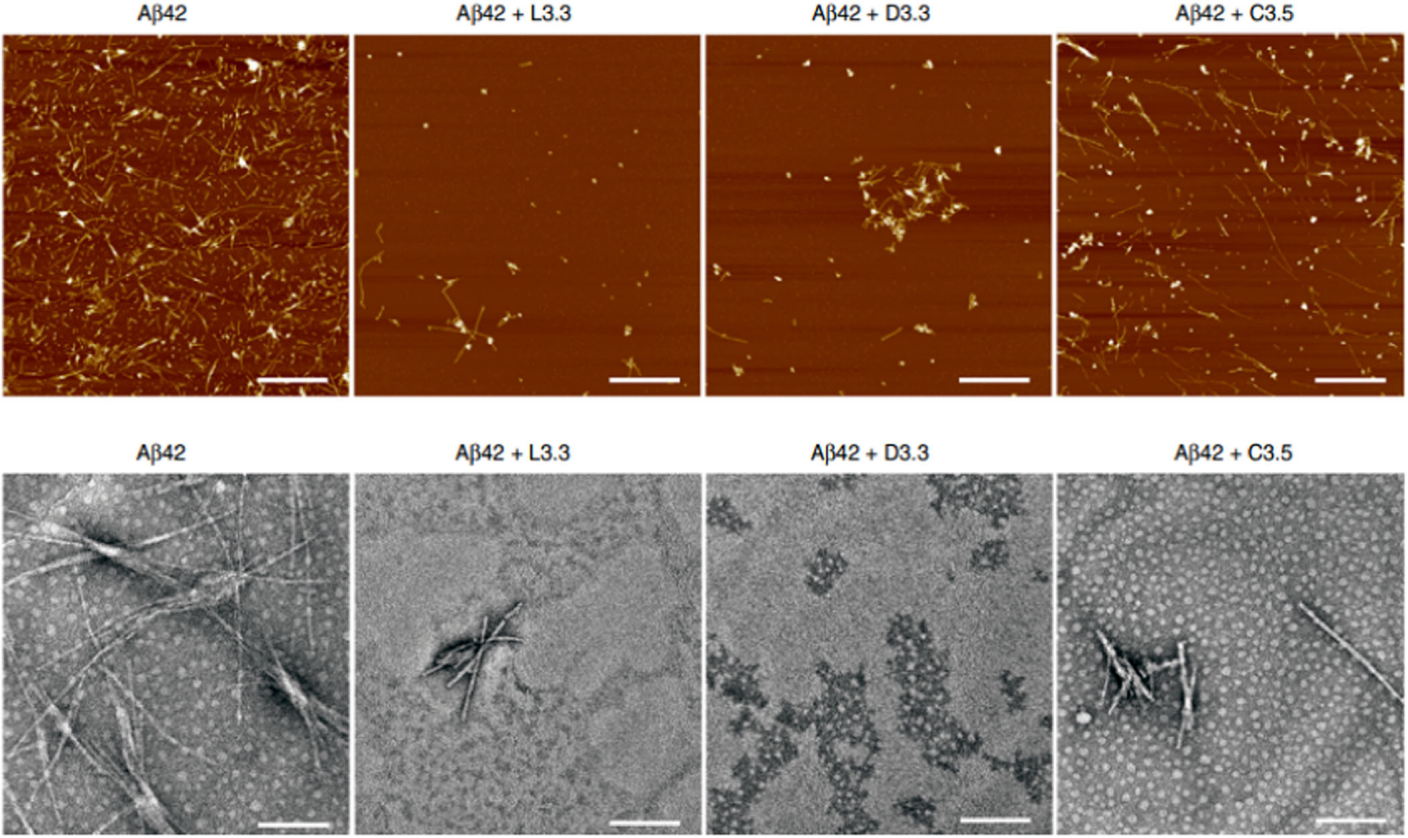
Top: atomic force microscopy (AFM) analysis of Aβ42 in the presence and absence of L3.3 and D3.3 after 48 h. down: transmission electron microscopy (TEM) in the presence and absence of L3.3 and D3.3 after 48 h.^78^ [Color figure can be viewed at wileyonlinelibrary.com]

In a recent study published in Nature Communication,^78^ very small‐size AuNPs (3.3 nm) were used as potential anti‐Aβ therapeutics. The peculiarity of this study is the surface functionalization, which occurred using the two chiral forms of glutathione (GSH), L and D, that are enantiomers forming Au nanoplatforms denominated L3.3 and D3.3, respectively. It is possible to enantio‐selectively inhibit the aggregation of Aβ in vitro. The comparison was made with citrate‐coated Au NPs which did not possess enantiomeric properties and therefore did not inhibit fibril aggregation (Figure 5). Furthermore, these nanostructures were demonstrated to overcome the BBB in mouse models and to act as a therapeutic agent to counteract AD.

**Figure 6:**
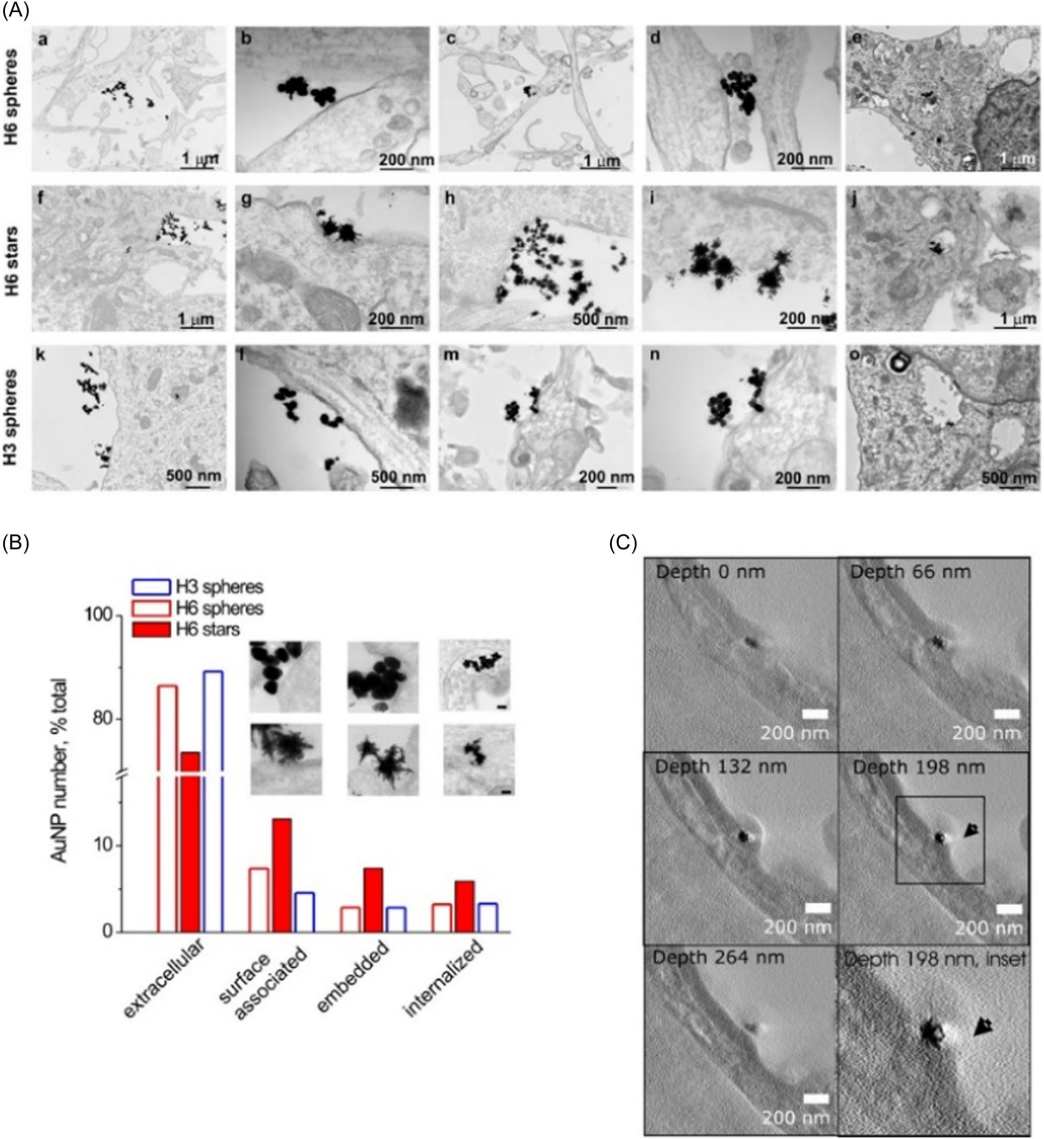
(A) Transmission Electron Microscopy (TEM) of Au spheres and stars functionalized by H3/H6 dendrimers for 24 H in hippocampal neurons at a concentration of 20 μg/mL quantitative assessment of Au nanostructures in different cell regions. (B) Quantification of AuNP distribution across cell compartments. (C) Orthoslices of neuron membrane at a depth of 66–198 nm with Au nanostars.^81^ [Color figure can be viewed at wileyonlinelibrary.com]

In addition to the antiaggregation action, it is possible to act on the side effects that the aggregation of amyloids induces, that is, the loss of cognitive abilities. This was possible, thanks to the use of animal models (rats) injected with Au NPs both by intraperitoneal and intrahippocampal injection. A comparison was made between citrate‐functionalized NPs and bucladesine‐functionalized NPs on a male Wistar rat model. The authors assessed the learning/memory‐related behavior. Furthermore, also the levels of Stromal interaction molecules 1 and 2 (STIM1 and STIM2) were examined after the administration of the AuNPs. These proteins are not physiologically expressed in AD models. Then, rats treated with AuNPs exhibited a general improvement in cognitive behavior due to their antiaggregating activity.^79^ The functionalization of NPs is a crucial point in the efficacy of therapy. PEG‐functionalized AuNPs loaded with anthocyanin were demonstrated to be neuro‐protective by inhibiting specific pathways targeting the Aβ plaques in the mice brains. The NPs crossed the BBB and exhibited a higher effect compared to the anthocyanins alone.^80^ Also, tuning the shape of AuNPs is possible to have different effects in neuroprotection also with different molecule functionalization, in particular, the bond of dendrimers named H3/H6 on spherical and star‐shaped NPs in hippocampal neurons. These nanostructures were able to cross a model BBB inducing the protection of neurons from oxidative stress and amyloid aggregation (Figure 6). In particular, the star‐shaped nanostructures induced greater effects than spherical NPs.^81^

Also, the synthesis method of nanomaterials can often affect the efficacy of NPs at the level of ND pathologies. For example, Au NPs produced from *Paeonia moutan* extracts were able to efficiently reduce ROS and cytokine production in vitro.^82^ Nontoxic phytochemicals are involved as reducing agents of metallic ions. To confirm the results, the NPs were tested in PD‐induced mice. In detail, C57BL/6 mice have been injected intraperitoneally with 30 mg/kg of 1‐methyl‐4‐phenyl‐1,2,3,6‐tetrahydropyridine hydrochloride (MPTP) for 5 consecutive days, which triggered a complete disruption of the DA neurons present in *substantia nigra* region of the brain. Upon incubation with AuNPs, prevention of neuroinflammation processes was observed with increasing dopamine levels. These mechanisms protected the mice from motor disturbances by restoring physiological behavioral situations. Biocompatible AuNPs with a size of 57.6 ± 3.07 nm were also obtained by *Ephedr sinica Stapf* to test them against neurodegenerative diseases, such as AD and PD. The authors observed decreased amount of neuroinflammatory cytokines that are commonly involved in the demyelination phenomenon. Also, ROS levels are reduced, making green AgNPs suitable as an anti‐inflammatory tool in the brain.^66^

Since plaque deposition is the principal phenomenon involved in brain neuropathy, Aβ aggregation triggers mitochondrial dysfunction that activates ROS production. The combination of Molybdenum disulfide (MoS_2_) with AuNPs, which is a typical transition metal chalcogenide, gave the nanotool the characteristics of an excellent absorption capacity under near‐infrared light irradiation. Then, the combination of NPs uptake and photothermal therapy can produce a synergistic effect on Aβ‐protein fibrils inhibiting the Aβ aggregation and reducing neurotoxicity. In addition, an improved cognitive and learning ability was observed in the AD mice model.^83^ The surface of AuNPs can be decorated with polymers such as polyoxometalate (POMD‐pep) to obtain a powerful tool capable to cross BBB and to inhibit the Aβ aggregation and reduce the Aβ‐mediated peroxidase activity.^84^ Dos Santos Tramontin et al.^85^ evaluated the effect in vivo of AuNPs (20 nm) in male Wistar rats treated with okadaic acid, a neurotoxin able to induce neurotoxicity. The experiments were performed with four experimental groups of rats, namely control, rats treated with AuNPs, OA, and a last group in which AuNPs and OA were administrated at the same time. As expected, the OA treatment induced a massive phosphorylation of τ protein and a reduction of neurotrophic factors, brain‐derived neurotrophic factor, and nerve growth factor‐β. Whereas, the animals exposed to AuNPs did not show this adverse effect. In addition, the prevention of oxidative stress was measured following the re‐establishment of superoxide dismutase and GSH levels activity in the brain. In addition, AuNPs prevented cognition impairment and mitochondrial dysfunction. Following the oxidative stress topic, and the molecules involved in the antioxidant cascade phenomena, glutathione‐functionalized AuNPs (GSH‐AuNPs) were demonstrated to be effective against Aβ aggregation in human neural stem cells (hNSCs). The mechanism was related to the decrease of the caspase 3 and 9 levels, which are involved in cell apoptosis. In addition to this, GSH‐AuNPs restored ATP amount by beneficial effects on cellular respiration, mitochondrial membrane potential, and mitochondrial genes.^86^

### 4.2 Detection of NDs by AuNPs

**Figure 7:**
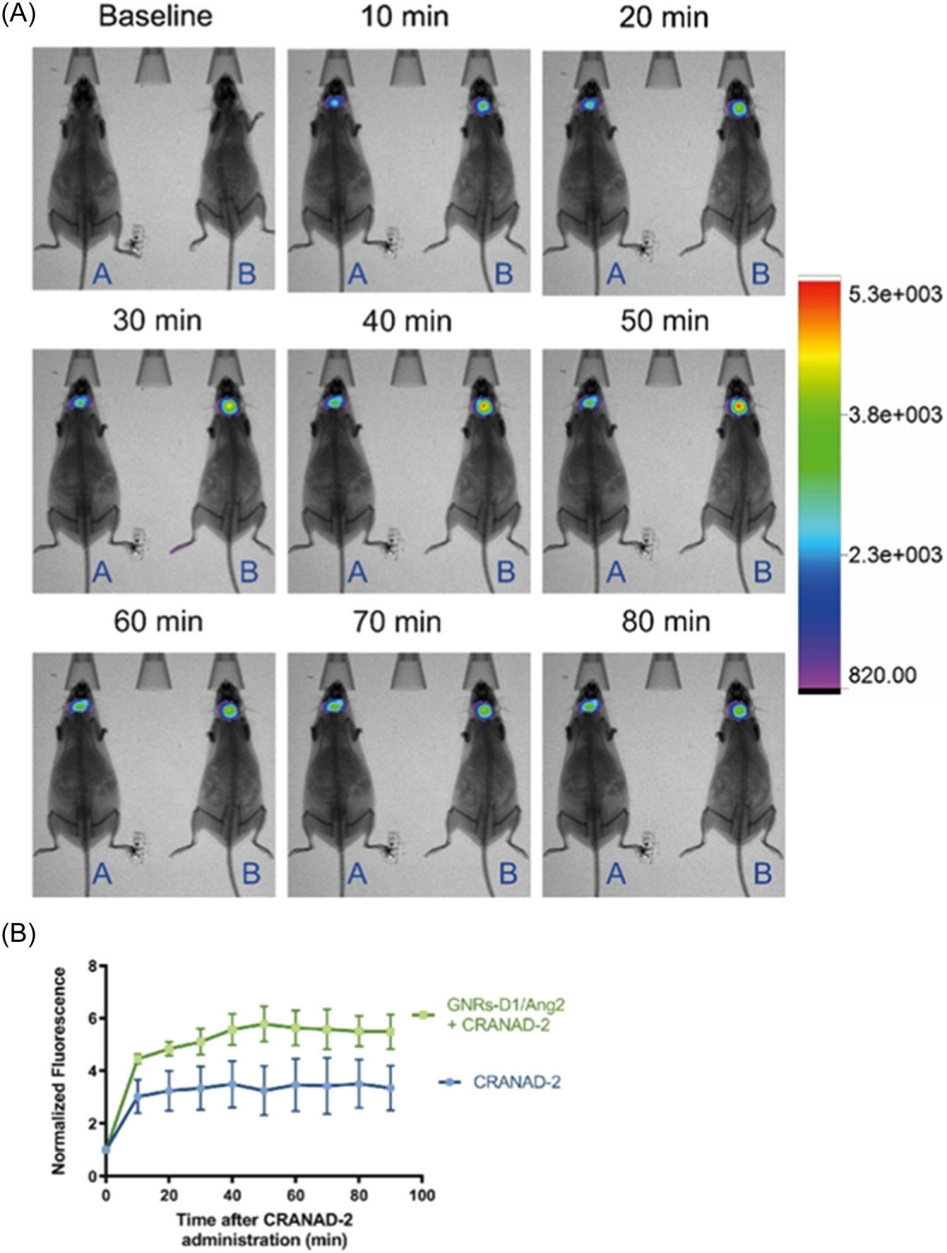
(A) Representative of APPS we/PSEN1dE9 TG mice incubated with CRANAD‐2 (A) or CRANAD‐2 + GNRs‐D1/Ang2 (B) using 640 nm excitation filter and 700 nm emission filter were used. (B) Normalized fluorescence intensity in the treated mice. Reprinted from Cabrera et al.,^90^ Copyright (2022), with permission from Elsevier. [Color figure can be viewed at wileyonlinelibrary.com]

In addition to using Au NPs as a therapeutic agent, they can be used as a contrast agent for brain imaging.^87^ The high atomic number of Au (*Z* = 79) makes AuNPs a better tool as contrast agents in medical imaging compared to iodine or gadolinium, which are characterized by high toxicity and can cause several side effects.^88^ A precise detection of Aβ accumulation, which is mandatory for the early diagnosis of AD, must be carried out within the near infrared window while investigating animal models.^89^ To this respect, the metal‐enhanced fluorescence of colloidal gold, namely the ability of noble nano‐colloids to enhance the emission signal of fluorescent dyes in the proximity of their surface, can overcome the intrinsic physicochemical limitations of standard fluorophores for in vivo applications. For this purpose, Au nanorods were chosen for their specific absorption in the infrared region. The detection of Aβ aggregates was demonstrated in a recent study, where Au nanorods have been functionalized with PEG and the fluorescent probes CRANAD‐2 or CRANAD‐58. These nanorods were able to selectively bind different amyloid species, and a strong imaging signal has been observed in both mouse and *C. elegans* in vivo models^90^ (Figure 7). The blood represents a suitable target for AD biomarkers, which include the Aβ 1–40, 1–42, and τ protein. However, their blood level is extremely low, making the exact measurements difficult with conventional techniques. This is why nanoplasmonic sensors, based on Au nanorods and guanidine hydrochloride as a chaotropic agent, have been designed and developed to detect AD in plasma samples.^91^ In particular, Au nanorods have been preliminarily functionalized with monoclonal antibody (mAb) to selectively recognize the AD biomarker. Therefore, upon binding the AD biomarkers with high affinity, a change in the resonance signal was generated, due to the SPR phenomenon triggered by Au in the context of the refractive index of the surrounding media. To improve the sensibility of the system, the authors used guanidine hydrochloride which is able to disrupt hydrophobic interactions between proteins, a process that enable the uncovering of the τ protein epitopes in the blood plasma. The mechanism induced by guanidine hydrochloride involves the breakup of hydrophobic interactions between proteins and water molecules^91^ (Figure 7B).

**Figure 8:**
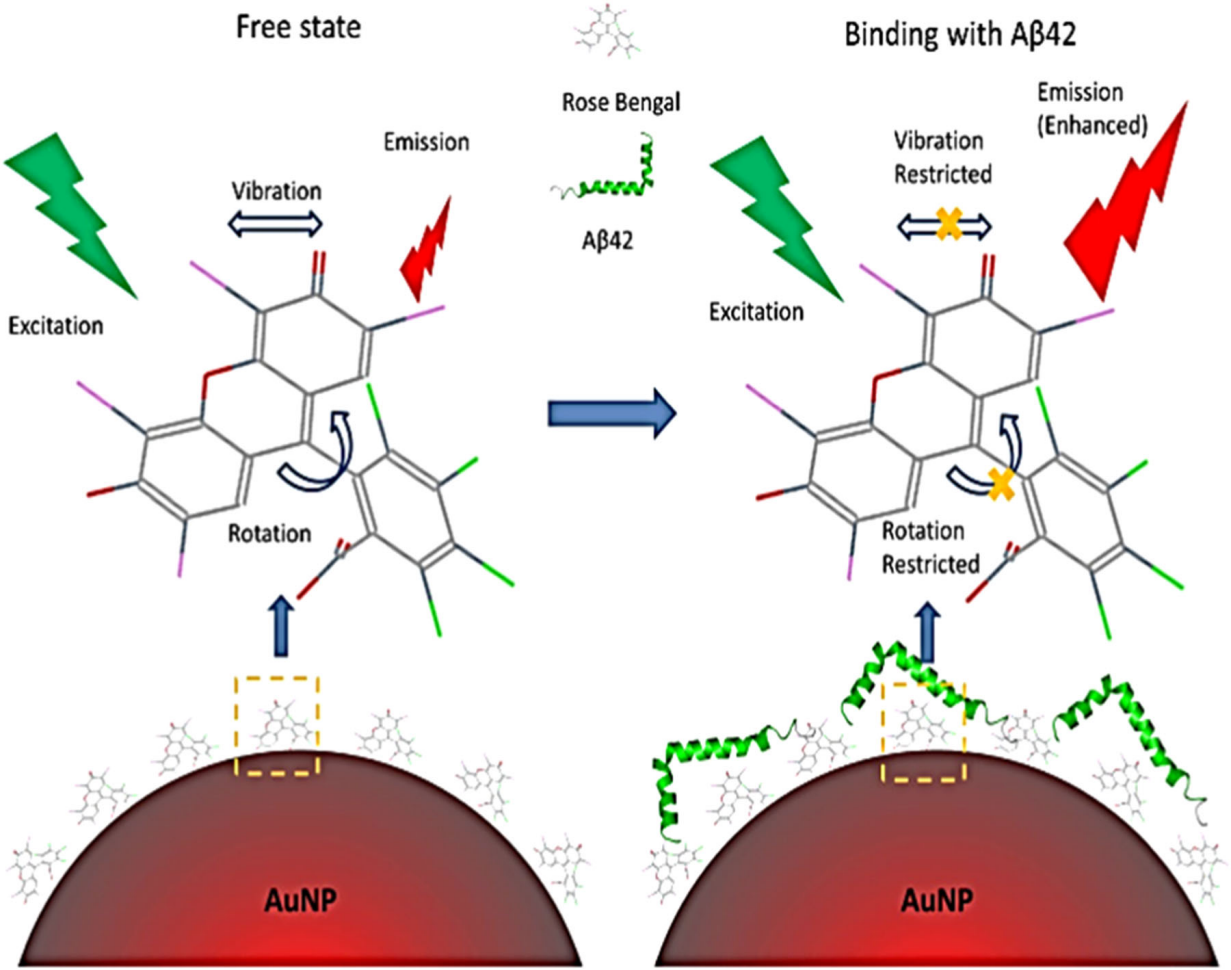
Schematic representation of AuNPs functionalized with RB and their interaction with Aβ. The emission was relative only to the NPs that selectively bind amyloids.^93^ Aβ, amyloid‐beta; AuNp, Gold nanoparticles; NP, nanoparticles; RB, Rose Bengal. [Color figure can be viewed at wileyonlinelibrary.com]

The Aβ‐oligomers in the brain can be identified, and potentially quantified, using AuNPs as optical probes based on the absorption intensity of plasmon resonance.^92^ As a matter of fact, the Aβ aggregates can be identified by quantifying the absorption shift (upon binding Aβ aggregates) using a combination of spectroscopic and microscopic techniques. This assay is simple, low‐cost, and label‐free. It may provide an attractive alternative for exploring the mechanistic properties of protein self‐assembly. It may also contribute to the development of nano‐based diagnostics for AD. Moreover, it is suitable to detect fibrillogenesis in neurodegenerative conditions. Aβ protein aggregates have been also detected by surface‐enhanced Raman scattering (SERS) probes. In particular, the conjugation of AuNPs with Rose Bengal (RB) dye was a suitable approach due to its great affinity with Aβ peptides. Upon binding, the Raman signal (from the probe) underwent a significant enhancement thanks to the SERS effect. The signal intensity was Aβ peptides dependent. In addition, authors noted a fluorescence increase of the RB which was used to produce images of amyloid plaques in mice brain samples to perform early diagnosis^93^ (Figure 8).

## 5 AgNPs

AgNPs have been extensively exploited in several biomedical applications.^94^ Similar to AuNPs, they exhibit SPR characteristics.^95^ They are also chemically stable and are widely used in commercial products, especially for their antibacterial properties against different types of bacteria, including antibiotic‐resistant ones.^96^ There are instead controversial data regarding their use in cancer treatment, due to their demonstrated indiscriminate toxicity toward healthy cells too. In fact, AgNPs tend to ionize upon internalization, causing the release of silver ions within the cell cytoplasm, activating the production of ROS, and causing mitochondrial damage. These pathways can then lead to cell death by apoptotic processes or necrosis.^97^, ^98^, ^99^ AgNPs have been demonstrated to reach the brain thanks to their intrinsic ability to cross the BBB,^100^ while their adsorption in the body usually happens through environmental exposure (like inhalation or skin contact).^101^

In addition to astrocytes, the brain is equipped with microglia, namely tissue‐resident macrophages that orchestrate the active immune defense against invading pathogens in the central nervous system. However, a chronic inflammatory state can induce side effects leading to the expression of TNF‐α, nitric oxide, and free radicals which, as previously reported, are involved in the impairment of the correct functioning of the microglia, followed by neuronal death typical of PD and AD.^102^, ^103^

**Figure 9:**
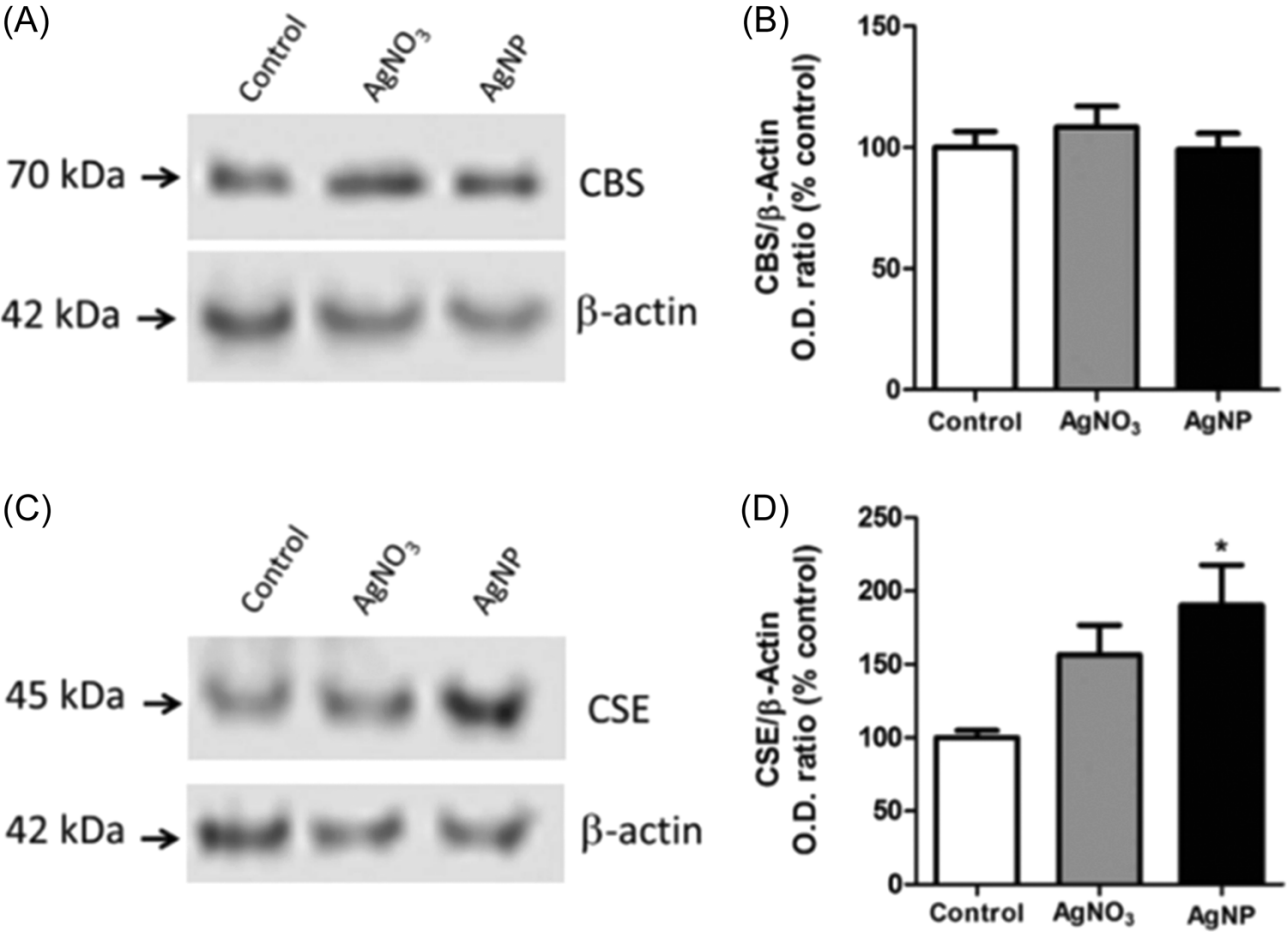
(A, C) AgNPs at a concentration of 50 µg/mL were used to incubate N9 microglia cells. In addition, also AgNO_3_ was used at a concentration of 3.5 µg/mL up to 24 h. Cystathionine β‐synthase (CBS) expression was quantified by Western blots after cell lysis. (B, D) The quantification of Western Blots analysis was assessed by enzyme optical density.^104^ AgNP, silver nanoparticle; CSE, cystathionine‐γ‐lyase.

In recent work,^104^ murine microglial N9 cell lines were used to investigate the effects of citrate‐capped AgNPs (50 nm, concentration 50 μg/mL). The authors tested the hypothesis whether AgNPs, upon entering the cells by endocytosis, can induce the expression of H_2_S‐synthesizing enzymes (cystathionine‐γ‐lyase, cystathionine β‐synthase, and mercaptopyruvate sulfurtransferase), triggered by the precipitation of silver ions to Ag_2_S. This process would lead to the formation of H_2_S which is a potent anti‐inflammatory molecule able to reduce the levels of lipopolysaccharide (LPS)‐induced ROS, nitric oxide, and TNF‐α production. H_2_S was demonstrated to reduce the effects of neuroinflammation also in DA neuronal cell line N27, which has been employed to examine whether modulation of microglial inflammation by AgNPs affects microglia‐mediated neurotoxicity (Figure 9).

However, AgNPs were proven to induce neurotoxicity following their accumulation in neuronal cells. This is why they are typically functionalized on their surface with suitable chemical molecules to be able to use as therapeutic agents against neurodegenerative diseases. Alternatively, it turned out that AgNPs produced through green and sustainable processes are less toxic and therefore can be used as therapeutic agents for neurodegenerative pathologies.^105^

For example, the leaf extract of *Nepenthes khasiana* ^106^ was employed as a reducing and capping agent for the synthesis of AgNPs, which were used in AD rat models induced by streptozotocin. The capping of polyphenols on the surface of the NPs was crucial to prevent deficits in recognition and spatial memory. Similarly, the leaf extract of mucin prurien, a leguminous plant native to Africa and Asia, has been used to produce 40 nm AgNPs.^107^ This plant is known for its high concentration of l‐dopa, which is an amino acid that stimulates the production of dopamine implicated in the treatment of Parkinson's. The authors evaluated the ability of AgNPs to counteract catalepsy, in a dose‐dependent manner, in healthy 3‐month‐old male mice, a typical condition of PD characterized by the loss of reaction to external stimuli.

The aqueous extracts of other plants, such as the *Lampranthus coccineus* and the *Malephora lutea* F. Aizoaceae, have been used to synthesize green AgNPs with a small size of ca. 3 and 30 nm, respectively.^108^ All the molecules constituted by the extracts were characterized to identify all the compounds. The AgNPs achieved by *L. coccineus* showed the highest antiacetylcholinesterase and antioxidant activity in rats in which AD was induced, compared to only phytocompounds. These results confirmed the ability of AgNPs to cross BBB and to increase the amount of acetylcholinesterase, reducing the oxidative stress, thanks to the presence of phyto molecules on the NPs' surface.

## 6 PERSPECTIVE AND FUTURE CHALLENGES

The use of nanomaterials for the diagnosis and treatment of neurodegenerative diseases is becoming a very influential topic in the scientific community. This is because, to date, there is no therapy capable of blocking the progression of neurodegenerative diseases. In fact, the therapies currently in use are based on prevention and early diagnosis.

In this review, we discussed the most recent publications concerning the use of Au and Ag‐based nanomaterials that have been shown to be very efficient in preventing amyloid plaques and oxidative stress. In addition, they can be used as powerful diagnostic tools. However, the real mechanism remains unclear, and above all the possible toxicity to the brain after the accumulation of NPs should be considered. However, to date, nanotechnologies appear to be a challenge for the eventual development of definitive therapy. It is important to note that the use of AuNPs and AgNPs for therapeutic purposes in neurodegenerative diseases is still at the experimental stage. Further research is needed to fully understand their mechanisms of action, optimize their properties, and evaluate their long‐term safety and efficacy. In particular, massive in vivo experiments should be performed to assess the possible toxic behavior of NPs and accumulation in other organs. Nonetheless, the potential of these NPs to prevent protein aggregation holds promise for future therapeutic interventions in neurodegenerative disorders.

## AUTHOR CONTRIBUTIONS

Valeria De Matteis conceived the idea of the manuscript. Valeria De Matteis and Edoardo Scarpa wrote the original draft. Mariafrancesca Cascione, Anna Griego, Paolo Pellegrino, and Giorgia Moschetti reviewed and edited the draft.

## CONFLICT OF INTEREST STATEMENT

The authors declare no conflicts of interest.

## ETHICS STATEMENT

Not applicable.

## Data Availability

Not applicable as no new data is generated.
